# DDX21 Enhances Radiosensitivity in Head and Neck Squamous Cell Carcinoma by Suppressing MK2‐Mediated DNA Damage Response

**DOI:** 10.1002/advs.77747

**Published:** 2026-09-16

**Authors:** Tianru Yang, Ting Shen, Yanfang Qiu, Wei Zhang, Ziran Zheng, Jiayi Sun, Yuedi Yao, Mianfeng Yao, Jiang Li

**Affiliations:** ^1^ Department of Oral Medicine Center of Stomatology Xiangya Hospital Central South University Changsha China; ^2^ Key Laboratory of Molecular Radiation Oncology Hunan Province Changsha China; ^3^ National Clinical Research Center for Geriatric Diseases (Xiangya Hospital of Central South University) Changsha China; ^4^ Xiangya Stomatological Hospital and Xiangya School of Stomatology Central South University Changsha China; ^5^ Hunan Key Laboratory of Oral Health Research Changsha China; ^6^ Department of Radiation Oncology The Affiliated Cancer Hospital of Xiangya School of Medicine Central South University Changsha China; ^7^ Department of Neurosurgery Xiangya Hospital Central South University Changsha China; ^8^ Department of Endodontics School and Hospital of Stomatology Guangzhou Medical University Guangzhou China

**Keywords:** DDX21, DNA damage response, HNSCC, MK2 inhibitor, radiotherapy sensitivity

## Abstract

Radioresistance remains a significant challenge in the radiotherapy (RT) of head and neck squamous cell carcinoma (HNSCC). However, the biological factors that govern sensitivity to this therapy are not well‐understood. The DEAD‐box family is known for its role in genome stability, and inextricably linked to the radiotherapy resistance of tumors. This study found the role of the RNA helicase DDX21 in regulating radiosensitivity through extensive data mining. High DDX21 expression predicted improved survival after postoperative radiotherapy. Overexpression of DDX21 increased radiosensitivity in vitro and in vivo, whereas depletion promoted radioresistance. In vitro, DDX21 enhanced radiation‐induced DNA damage, genomic instability, and apoptosis by binding MK2 and suppressing MK2 phosphorylation independently of p38 activity. Meanwhile MK2 inhibition restored and further augmented radiosensitivity in DDX21‐deficient cells and xenografts by increasing DNA damage and apoptosis. Overall, DDX21 regulates radiosensitivity in HNSCC by suppressing MK2 signaling and modulating the radiation‐induced DNA damage response. Its expression may serve as a potential biomarker associated with radiosensitivity, and MK2 inhibition offers a promising approach to overcome radioresistance in tumors with low DDX21 expression.

## Graphical abstract

DDX21 suppresses MK2 activation by directly inhibiting Thr334 phosphorylation in a p38‐independent manner, thereby impairing DNA damage response. Clinically, patients with well‐differentiated tumors exhibiting high DDX21 expression are more likely to benefit from adjuvant radiotherapy, whereas those with low DDX21 levels are predisposed to radioresistance. The figure was created using Figdraw (www.figdraw.com).

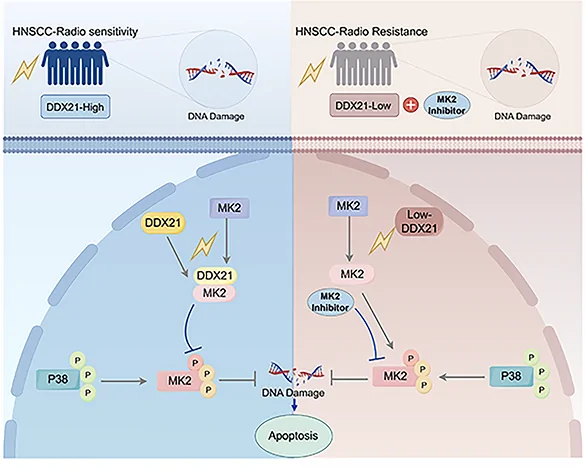

## 1 Introduction

Head and neck squamous cell carcinoma (HNSCC) remains a leading cause of cancer mortality worldwide, with an estimated 1.08 million new cases projected annually by 2030 [1]. The standard of care for locally advanced HNSCC typically involves surgery followed by adjuvant chemoradiotherapy (CRT), whereas radiotherapy serves as the primary curative modality for patients with unresectable disease [2]. Despite these established therapeutic approaches, clinical outcomes remain unsatisfactory; more than 65% of patients eventually experience recurrence or metastasis, leading to a 5‐year overall survival (OS) rate of less than 50% [3]. Current prognostic indicators, such as tumor stage and histological grade, offer limited accuracy in predicting individual radioresistance. Furthermore, effective therapeutic to reverse radioresistance remain elusive [4]. These challenges highlight a pressing need to identify novel regulators of radiosensitivity in HNSCC and to establish their potential as clinically actionable biomarkers or therapeutic targets.

Aberrations in DNA damage response (DDR) pathways play a critical role in shaping the radiosensitivity of HNSCC. A retrospective analysis of 170 patients revealed *BRCA2* and *ARID1A* as the most frequently mutated DDR genes, with recurrent alterations also detected in *ATM* and *BRCA1 [* 5, 6]. In addition, sequencing of circulating tumor DNA from 75 patients identified DDR‐associated mutations, including *APC*, *ATM*, and *BRCA1/2*, in 38.8% of cases [7]. Clinically, reduced expression of *ATM* or *BRCA1* has also been linked to poorer OS and worse prognosis in clinical studies [8]. These observations underscore the importance of DDR pathways in modulating radiotherapy response and suggest that targeting DDR mechanisms may offer a promising strategy to improve therapeutic efficacy.

Although the core components of DDR pathways in HNSCC have been extensively characterized, growing evidence indicates that RNA‐processing factors, particularly RNA helicases, significantly modulate DDR efficiency through underrecognized mechanisms [9, 10]. The DEAD‐box family of RNA helicases is known for its role in genome stability. These DEAD‐box helicases constitute a major class of ATP‐dependent enzymes that govern RNA binding, unwinding, and structural remodeling during diverse RNA metabolic processes [11, 12]. Beyond these canonical functions, they have emerged as pivotal regulators of cancer progression and therapeutic responsiveness by regulating DNA damage repair, replication stress, and cell cycle control [13, 14]. DEAD‐box RNA helicase 21 (DDX21), a key member of the 37‐protein DEAD‐box family, has been implicated in the direct regulation of multiple DDR processes [15]. In colorectal cancer, elevated DDX21 expression has been shown to delay homologous recombination repair and increase replication stress, thereby impairing DDR function and promoting genomic instability [16]. Moreover, DDX21 contributes to the maintenance of genome integrity by resolving transcription‐associated R‐loops and preventing R‐loop‐induced DNA damage [17].

Recent studies have consistently highlighted the critical role of DDX21 in cancer biology, supporting its potential as both a biomarker and a therapeutic target, while also revealing its context‐dependent oncogenic or tumor‐suppressive functions. DDX21 promotes gastric tumorigenesis by preventing its ubiquitin‐mediated degradation [18], drives breast cancer proliferation through ribosome biogenesis, and supports acute myeloid leukemia by activating autophagy‐related pathways [19]. In colorectal cancer, DDX21 promotes metastasis by activating an MCM5‐dependent epithelial‐to‐mesenchymal transition pathway [16]. These findings underscore the diverse and context‐specific roles of DDX21 across malignancies. However, its mechanistic contribution to radiotherapy response in HNSCC and its clinical significance remain poorly understood.

This study investigates the role of DDX21 in regulating the radiotherapy response of HNSCC. Through integrated analyses of clinical samples and in vivo xenograft models, we demonstrate that higher DDX21 expression is significantly associated with improved overall survival following postoperative radiotherapy, supporting its potential as a prognostic biomarker. Mechanistically, DDX21 enhances radiation‐induced DNA damage and promotes apoptosis by binding to and suppressing MAPK‐activated protein kinase 2 (MK2) activity. This DDX21–MK2 interaction amplifies genotoxic stress and modulates radiosensitivity. Notably, pharmacologic inhibition of MK2 markedly increased radiosensitivity in DDX21‐deficient HNSCC cells and xenografts, highlighting a promising therapeutic strategy for overcoming radioresistance. These findings establish DDX21 as a clinically meaningful biomarker and reveal the DDX21–MK2 axis as a viable target for improving radiotherapy outcomes in HNSCC. These insights provide a foundation for developing more effective and personalized radiotherapy approaches for patients with HNSCC.

## 2 Results

### 2.1 DEAD‐Box Family Member DDX21 as the Key Regulator of Radiosensitivity in HNSCC

**FIGURE 1:**
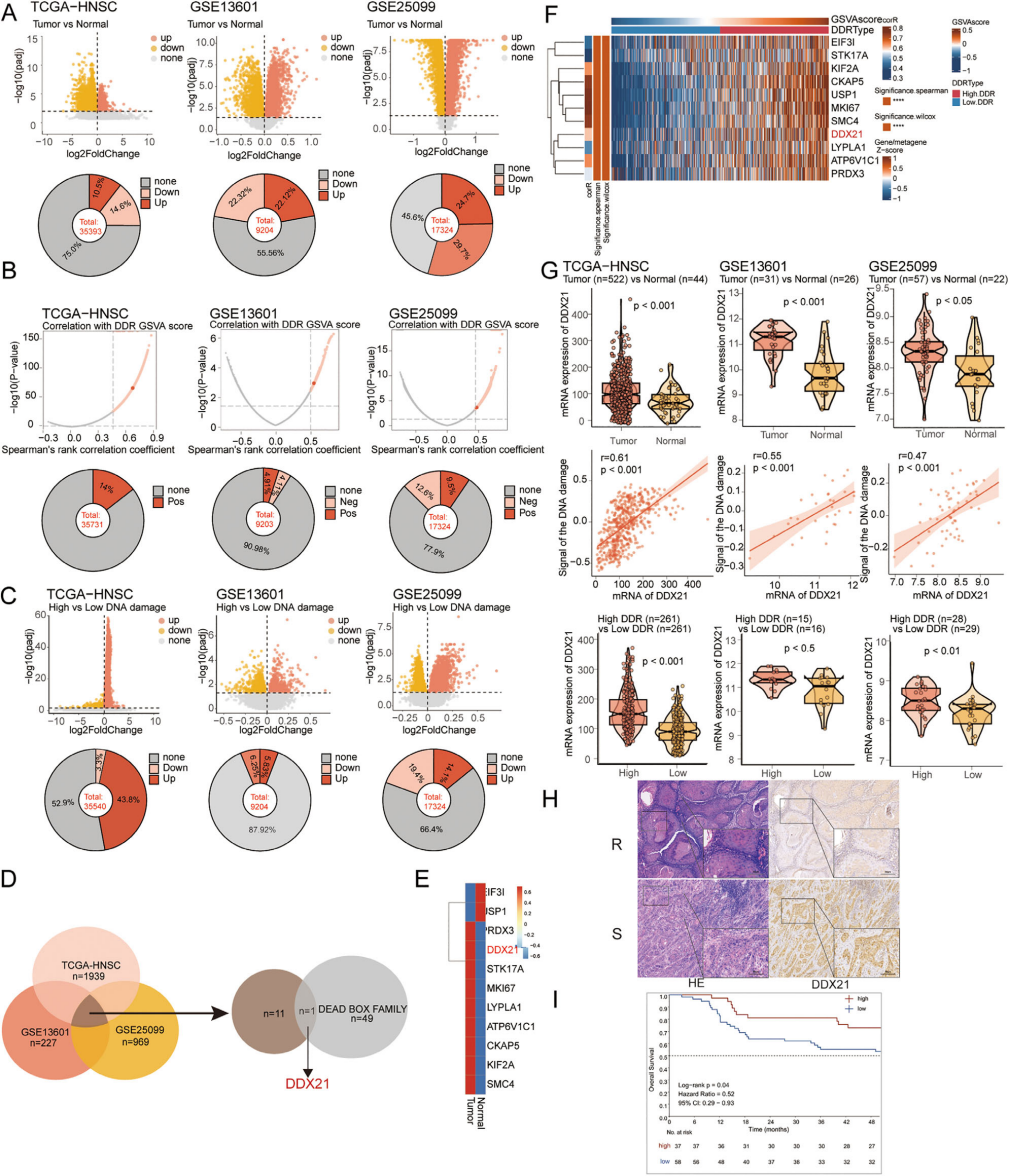
DDX21 as a core DEAD‐box helicase regulating radiosensitivity in HNSCC. A. Volcano plots and pie charts showing DEGs between HNSCC tumor and normal tissues across the TCGA, GSE13601, and GSE25099 datasets. B. Scatter plots and pie charts displaying genes significantly correlated with DDR pathway enrichment scores (GSVA) in the three datasets. C. Volcano plots and pie charts showing DEGs between patients stratified into high‐ and low‐DDR score groups. D. Venn diagram illustrating the screening strategy: the intersection of significant genes from A, B, and C yielded 11 candidates, which were further intersected with the DEAD‐box family to identify DDX21. E. Heatmap summarizing the differential expression levels of the 11 candidates in tumor versus normal tissues. F. Heatmap visualizing the expression patterns of the 11 overlapping DDR‐related genes identified in D. (Blue: low expression; Red: high expression). G. Validation of DDX21 across three cohorts (TCGA, GSE13601, GSE25099). Top: Violin plots of DDX21 mRNA expression in tumor versus normal tissues. Middle: Scatter plots showing the correlation between DDX21 expression and DDR scores. Bottom: DDX21 expression levels in high‐ versus low‐DDR score groups. H. Representative IHC images of DDX21 in radiosensitive versus radioresistant HNSCC tissues. I. Kaplan‐Meier survival curve comparing overall survival between HNSCC patients with high (red) and low (blue) DDX21 expression receiving postoperative radiotherapy. (Data are presented as mean ± SD. *p < 0.05, **p < 0.01, ***p < 0.001).

A multi‐cohort transcriptomic analysis was conducted to identify DEAD‐box RNA helicases associated with the radiation response in HNSCC. Differential expression analysis in TCGA (522 tumors vs 44 normal tissues) identified 8,862 differentially expressed genes (DEGs), while 4049 and 9426 DEGs were identified in GSE13601 and GSE25099, respectively (Figure 1A) [20, 21, 22]. Using the DDR pathway scores derived from GSVA and combined with Spearman correlation analysis, DDR‐related genes were identified in three datasets: 5177 in the TCGA‐HNSC dataset, 452 in the GSE13601 dataset, and 1645 in the GSE25099 dataset (Figure 1B). Subsequently, HNSCC patients were stratified into high‐ and low‐DDR subgroups based on the median DDR pathway score, and subgroup‐specific DEGs were identified. In the TCGA‐HNSC cohort, 15 562 genes were upregulated in the low‐DDR subgroup compared with the high‐DDR subgroup (43.8%), with corresponding numbers of 537 (5.83%) and 2,449 (14.1%) in the GSE13601 and GSE25099 cohorts, respectively. Conversely, 1175 genes were downregulated in TCGA‐HNSC (3.3%), as well as 575 (6.25%) in GSE13601 and 3369 (19.4%) in GSE25099 (Figure 1C). Within each cohort, the intersection of upregulated and downregulated DEG lists yielded 1,939, 227, and 969 genes in TCGA‐HNSC, GSE13601, and GSE25099, respectively. Intersection of these datasets yielded 11 shared DDR‐related genes (Figure 1D). Most candidate genes such as DDX21, STK17A, MKI67, and CKAP5 displayed significantly higher expression in tumor tissues than those in normal tissues. Among these, *DDX21* was the only member overlapped with the DEAD‐box gene family, nominating it as the primary candidate (Figure 1E,F). Further analysis of the main candidate gene *DDX21* revealed a significant increase in its expression in tumor tissues across all cohorts, and positively correlated with the DDR pathway score (Figure 1G). To validate these findings, IHC staining of 21 paired HNSCC and adjacent non‐tumor tissues confirmed higher DDX21 protein levels in tumors, predominantly localized to the nucleus (Figure S2A,B). Consistently, *DDX21* mRNA levels were significantly increased in 14 paired fresh tumor samples (Figure S2C). In vitro experiments revealed that DDX21 promotes the proliferation of tumor cells, but has no effect on metastasis (Figure S2D–G).

To determine the clinical relevance of DDX21 in radiotherapy response, IHC staining was performed on tumor specimens from 97 HNSCC patients who received postoperative radiotherapy. Patients were classified into radiosensitive (*n* = 48) and radioresistant (*n* = 47) groups based on treatment outcomes. DDX21 expression was markedly elevated in radiosensitive tumors and was significantly associated with well‐differentiated tumors. Kaplan‐Meier overall survival (OS) curves showed that patients with high expression had significantly better survival outcomes compared with the low‑expression group (Figure 1H,I, Table 1). In contrast, no significant association between DDX21 expression and overall survival was observed in TCGA‑HNSCC patients, suggesting that the prognostic value of DDX21 is specific to radiotherapy‑treated cohorts. (http://gepia.cancer‐pku.cn/detail.php?gene = DDX21). These data indicate that DDX21 is closely associated with radiosensitivity and represents a potential biomarker associated with radiosensitivity in HNSCC.

**TABLE 1:** Association between DDX21 IHC expression and clinicopathological characteristics in HNSCC patients receiving postoperative radiotherapy.

Variables		IHC score		*Χ* ^2^	*P*
		Low(*n*)	High(*n*)		
Age (years)	< 60	50	30	0.538	0.464
	≥ 60	9	8		
Gender	Male	52	35	—	0.736^a^
	Female	7	3		
Tumor size	T1‐T2	42	26	0.0843	0.772
	T3‐T4	17	12		
Lymph node metastasis (2 cases excluded)	N0	20	11	0.391	0.532
	N1‐N3	37	27		
Pathological grade	Well differentiated	25	25	5.071	0.024
	Moderately/poorly differentiated + undifferentiated	34	13		
5‐year overall survival(2 cases excluded)	Survived	24	24	4.994	0.025
	Not survived	34	13		
p16	Positive	4	3	—	1.000^a^
	Negative	55	35		

*Note*: Categorical variables were analyzed using the χ^2^ test.

^a^Two‑tailed Fisher's exact test was applied for tables with expected cell counts <5.

Given the incomplete and heterogeneous annotation of HPV status in TCGA‐HNSCC, we restricted the HPV‐stratified analysis to the GSE65858 cohort [23, 24]. HPV‐positive tumors exhibited higher DDR scores but lower DDX21 expression than HPV‐negative tumors (Figure S2H,I). Nevertheless, DDX21 expression remained positively correlated with the DDR score within both HPV subgroups, indicating that the association between DDX21 expression and DDR activity was preserved after stratification by HPV status (Figure S2J,K).

We further evaluated the relationship between DDX21 expression and p16 status in an independent clinical cohort of 97 patients with HNSCC who received postoperative radiotherapy and had available p16 status. Immunohistochemical analysis identified 7 p16‐positive and 90 p16‐negative tumors. The distribution of p16 status did not differ significantly between the DDX21‐low and DDX21‐high groups (Table 1). For survival analysis of the p16‐negative subgroup, two patients were excluded because of incomplete follow‐up information, leaving 88 evaluable p16‐negative cases. Consistent with the findings in the overall cohort, high DDX21 expression remained associated with improved overall survival in both the entire cohort and the p16‐negative subgroup (Figure S2L,M). These findings suggest that the prognostic association of DDX21 expression was not solely attributable to differences in p16 status and remained evident in the predominantly p16‐negative population.

### 2.2 DDX21 Exacerbates DNA Damage and Genomic Instability in Irradiated HNSCC Cells

**FIGURE 2:**
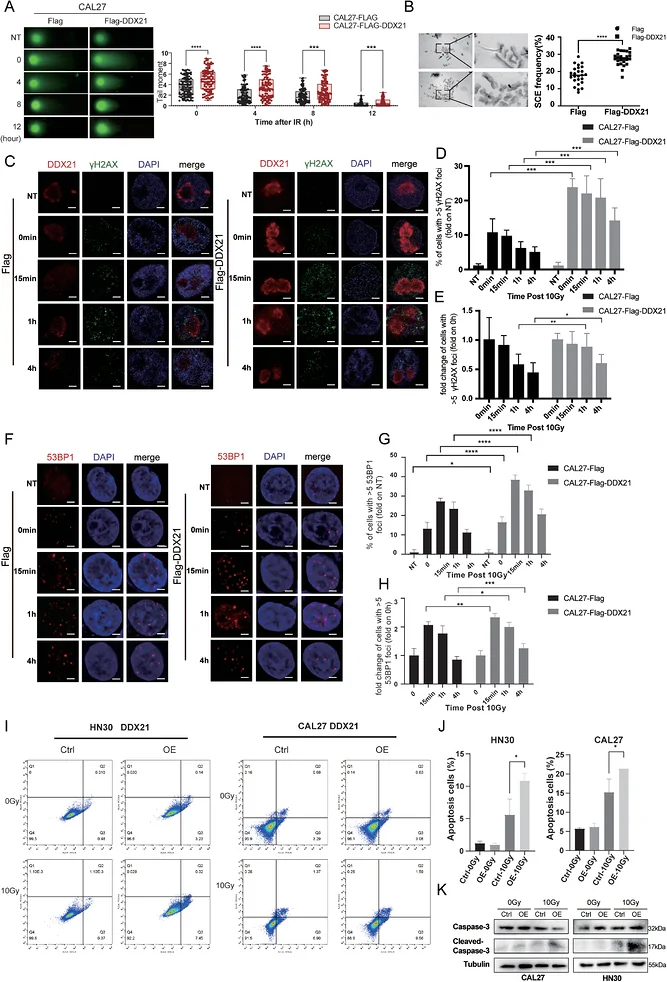
DDX21 overexpression increases radiation‐induced DNA damage and enhances radiosensitivity in HNSCC cells. A. Representative neutral comet assay images and quantification of tail moments in CAL27 cells expressing Flag or Flag‐DDX21 at 0, 4, 8, 12 h after exposure to 10 Gy irradiation (*n* = 100; error bars = ± SEM). B. Representative images of metaphase chromosomes (left; arrows indicate sister chromatid exchanges, SCEs) and statistical quantification of SCE frequency (right) in CAL27 cells with control (DDX21‐Ctrl) or *DDX21* overexpression. C. Immunofluorescence detection of γH2AX foci in CAL27 cells stably expressing vector control or DDX21 at 0 min, 15 min, 1 h, and 4 h following 10 Gy irradiation (scale bar = 5 µm). D, E. Quantitative analysis of γH2AX foci per nucleus at each time point. D. Fold change in the proportion of cells with >5 γH2AX foci relative to their respective non‐treated (NT) controls. E. Fold change in the proportion of cells with >5 γH2AX foci relative to their respective 0 h values after IR. F–H. Quantitative analysis of 53BP1 foci per nucleus at each time point in CAL27 cells. F. Representative 53BP1 immunofluorescence images of control (Flag) and DDX21‐overexpressing (Flag–DDX21) CAL27 cells at the indicated times after 10 Gy irradiation. Scale bars, 5 µm. G. Fold change in the proportion of cells with >5 53BP1 foci relative to their respective non‐treated (NT) controls. H. Fold change in the proportion of cells with >5 53BP1 foci relative to their respective 0 h values after IR. I. Flow cytometric analysis of apoptosis in CAL27 and HN30 cells overexpressing *DDX21* or vector control after 10 Gy irradiation. J. Quantification of apoptotic cell percentages corresponding to panel I. K. Western blot analysis showing expression of cleaved caspase‐3 in CAL27 and HN30 cells with stable overexpression of empty vector or DDX21 after 10 Gy irradiation. (*p < 0.05, **p < 0.01, ***p < 0.001.).

We next investigated the role of DDX21 in the DNA damage response induced by IR in HNSCC. CAL27 cells stably overexpressing DDX21 or corresponding control vectors were exposed to 10 Gy IR (Figure S1B). Neutral comet assays showed higher tail moments in DDX21‐overexpressing cells at 0, 4, 8, and 12 h after IR, indicating an increased DSB burden throughout the post‐irradiation observation period (Figure 2A and Figure S3A,B). SCE assays further showed a significant increase in SCE events in DDX21‐overexpressing CAL27 cells compared with controls (Figure 2B), reflecting enhanced chromosomal instability. Consistently, immunofluorescence analysis further revealed increased IR‐induced γH2AX and 53BP1 foci across multiple post‐irradiation time points (Figure 2C–H and Figure S3C–E). We additionally assessed basal chromatin organization in DDX21‐overexpressing cells. Following MNase digestion, DDX21‐overexpressing cells displayed increased mononucleosomal DNA fragments together with reduced high‐molecular‐weight DNA fragments, accompanied by decreased H3K9me3 levels (Figure S4). These observations prompted us to investigate whether the enhanced DNA damage induced by DDX21 overexpression ultimately resulted in increased apoptotic cell death following irradiation. Flow cytometric analysis using Annexin V‐FITC staining revealed that, under basal conditions, apoptotic rates were comparable between DDX21‐overexpressing and control cells; however, following IR, apoptosis markedly increased, with a higher proportion of apoptotic cells observed in the DDX21‐overexpressing group (Figure 2I,J). Correspondingly, levels of cleaved caspase‐3 were elevated after IR in both CAL27 and HN30 cells overexpressing DDX21 (Figure 2K). These findings demonstrate that DDX21 amplifies IR‐induced DNA damage, promotes genomic instability, and enhances apoptosis in HNSCC cells.

### 2.3 DDX21 Enhances Radiosensitivity of HNSCC In Vivo and In Vitro

**FIGURE 3:**
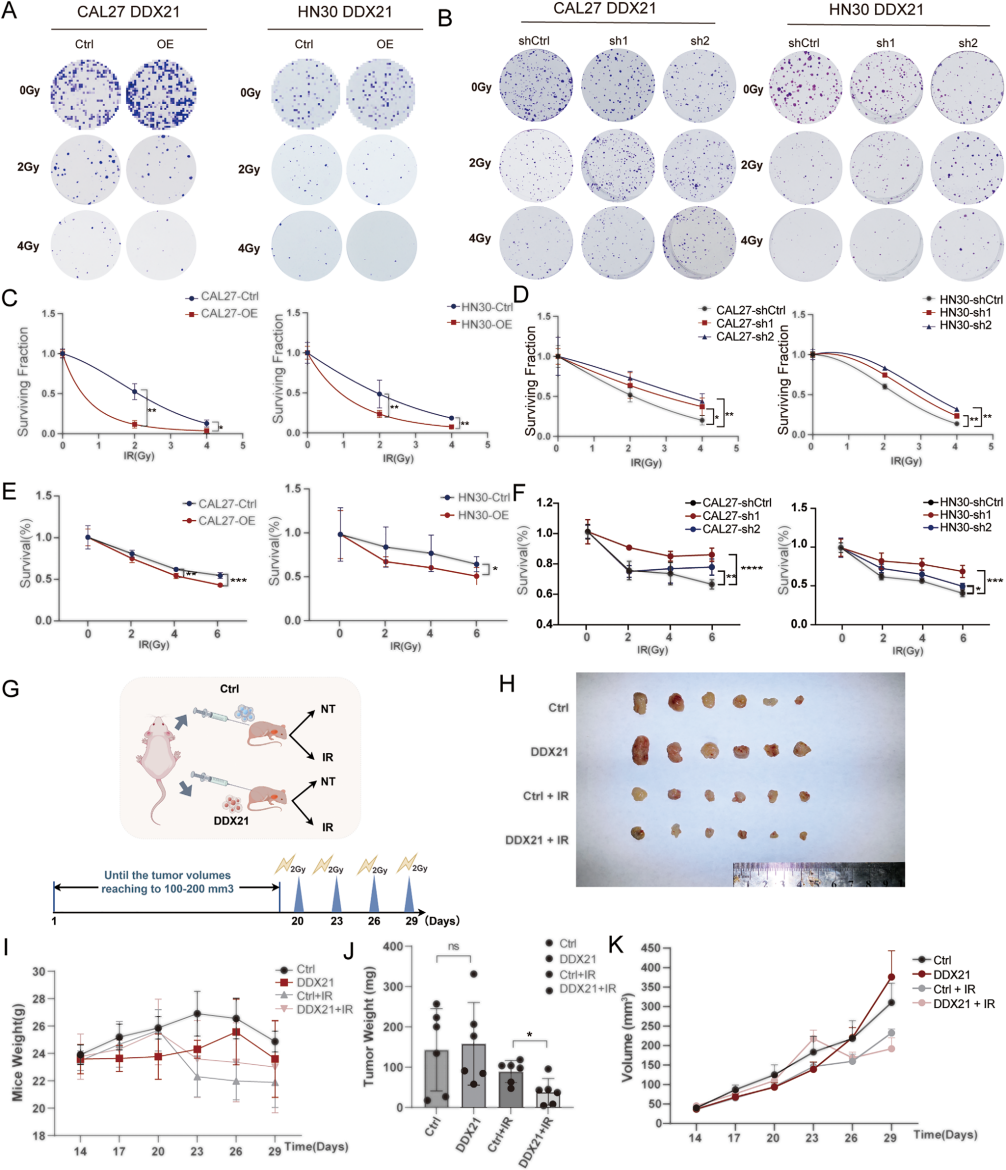
DDX21 enhances radiosensitivity of HNSCC in vivo and in vitro. A, C. Colony formation assays of CAL27 and HN30 cells with stable *DDX21* overexpression or vector control following irradiation at 0, 2, or 4 Gy, assessed 21 days post‐treatment. A shows representative images and C shows the quantitative surviving fraction. B, D. Colony formation assays of CAL27 and HN30 cells with stable *DDX21* knockdown or vector control subjected to the same irradiation doses and evaluated 21 days later. B shows representative images and D shows the quantitative surviving fraction. E. Cell viability of CAL27 and HN30 cells overexpressing *DDX21* or vector control after exposure to 0, 2, 4, or 6 Gy, measured by CCK‐8 assay 3 d post‐irradiation. F. Cell viability of CAL27 and HN30 cells with *DDX21* knockdown or vector control following irradiation at 0, 2, 4, or 6 Gy, assessed by CCK‐8 assay 3 days after treatment. G, H. Representative images of xenograft tumors derived from CAL27 cells with vector control, *DDX21* overexpression, vector plus IR, or *DDX21* overexpression plus IR. Tumors were collected and measured on day 29. The figure was created using Figdraw (www.figdraw.com). I. Weight changes of nude mice inoculated with overexpression control or DDX21 stable cell lines following IR treatment. J. Tumor weights in each xenograft group (n = 6; mean ± SD; *p < 0.05). K. Tumor growth curves showing xenograft volume changes over the course of treatment in each group.

We next assessed the impact of DDX21 on radiosensitivity in HNSCC. CAL27 and HN30 cells with stable *DDX21* overexpression or knockdown were exposed to graded doses of IR. Colony formation and CCK‐8 assays revealed that *DDX21* overexpression markedly reduced clonogenic survival (Figure 3A,C), and CCK‐8 assays revealed that *DDX21* overexpression reduced cell viability following irradiation (Figure 3E). In contrast, *DDX21* knockdown significantly increased colony formation (Figure 3B,D) and enhanced cell viability at corresponding radiation doses (Figure 3F). To validate these findings in vivo, we generated a nude mouse xenograft model using CAL27 cells. Although irradiation suppressed tumor growth across all groups relative to non‐irradiated controls (Figure 3G,H), tumors derived from DDX21‐overexpressing cells exhibited substantially smaller volumes and weights after irradiation compared with vector controls (Figure 3J,K). Even though the mice in the treatment groups exhibited a slight decrease in body weight, this change is attributable to the expected side effects of ionizing radiation (Figure 3I). These results indicate that DDX21 enhances HNSCC radiosensitivity in vivo and in vitro.

### 2.4 Exploring the Role of DDX21 in the Inhibition of MK2 Kinase Activity

**FIGURE 4:**
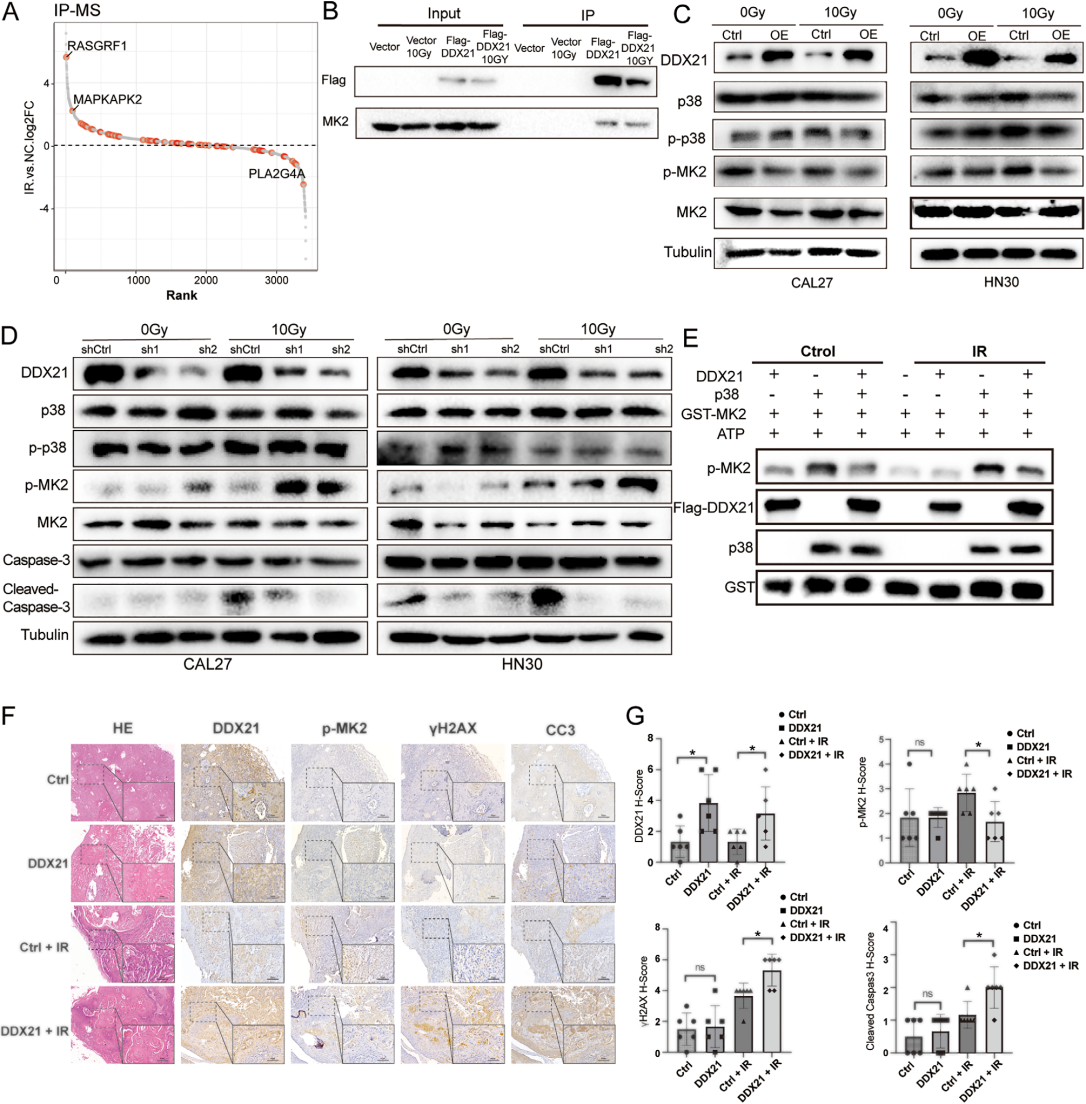
DDX21 inhibits MK2 kinase activity in response to irradiation. A. Co‐immunoprecipitation (Co‐IP) combined with mass spectrometry (MS) identifying differentially interacting proteins in irradiated CAL27 cells overexpressing *DDX21* compared with control cells. Key interaction candidates are shown. B. Co‐IP validation of the interaction between DDX21 and MK2, demonstrating enhanced binding following irradiation. C. Immunoblot analysis of p38 pathway components and apoptosis‐related proteins in CAL27 and HN30 cells with stable *DDX21* overexpression or vector control after 10 Gy irradiation. D. Immunoblot analysis of the same protein panel in CAL27 and HN30 cells with stable *DDX21* knockdown or vector control following 10 Gy irradiation. E. In vitro kinase assay demonstrating that DDX21 suppresses MK2 phosphorylation independently of p38 kinase activity. F. Representative immunohistochemical staining for DDX21, p‐MK2, γH2AX, and cleaved caspase‐3 in xenograft tumor sections (scale bar = 50 µm). G. Quantitative scoring of immunohistochemical staining for each protein (*n* = 6; mean ± SD; *p < 0.05).

To explore the potential molecular mechanism of DDX21 in HNSCC, we performed Co‐IP/MS on irradiated DDX21‐overexpressing CAL27 cells (Figure 4A). Mass spectrometry analysis identified a marked increase in MK2, a key component of the MAPK pathway, following IR. Subsequent Co‐IP experiments confirmed that the interaction between DDX21 and MK2 was substantially strengthened after irradiation (Figure 4B). The p38 MAPK pathway is a critical determinant of cell fate following IR. While previous studies have reported a cytoprotective role of p38 MAPK in the radiation response [25, 26], others have suggested a minimal or negligible contribution [27], indicating that the putative function of p38 MAPK in radiotherapy remains to be fully clarified. Because MK2 is activated through Thr334 phosphorylation (p‐MK2) by p38 [28], we hypothesized that DDX21 may compete with p38 for MK2 binding, thereby suppressing MK2 activation. To test this hypothesis, *DDX21* was either overexpressed or silenced in CAL27 and HN30 cells. Following irradiation, p‐MK2 levels were reduced in *DDX21*‐overexpressing cells but increased upon *DDX21* knockdown, while total MK2, p38, and p‐p38 levels remained unchanged (Figure 4C,D and Figure S5). Furthermore, in vitro kinase assays demonstrated that DDX21 directly inhibited MK2 phosphorylation without affecting the kinase activity of p38 (Figure 4E). In nude mouse xenograft model, immunohistochemical analysis further demonstrated reduced p‐MK2 expression and increased γH2AX and cleaved caspase‐3 levels in DDX21‐overexpressing tumors (Figure 4F,G). These findings indicate that DDX21 binds to MK2 and suppresses its phosphorylation independently of p38 activity, establishing DDX21 as a negative regulator of MK2 signaling in HNSCC.

### 2.5 MK2 Inhibitor Sensitizes *DDX21*‐Deficient HNSCC Cells to Radiotherapy

**FIGURE 5:**
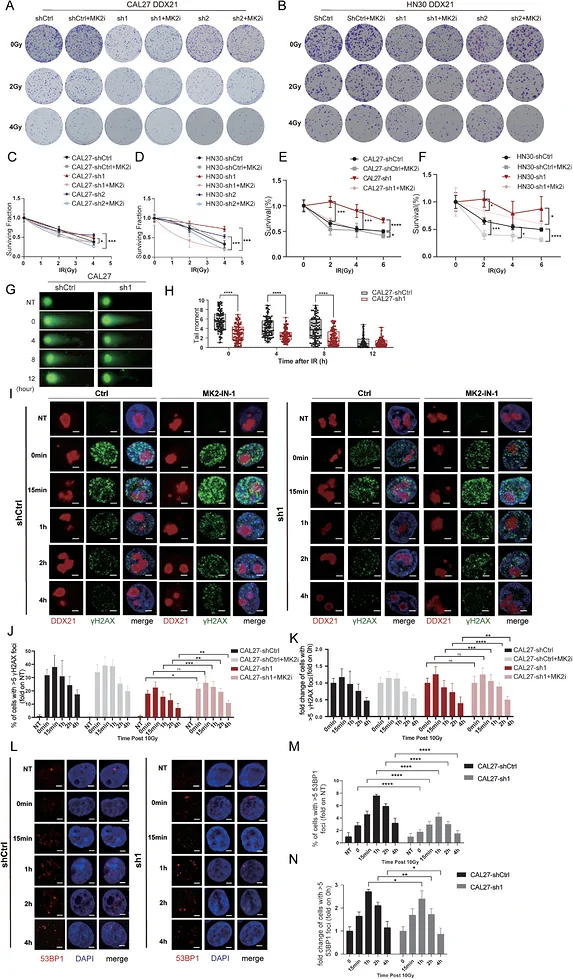
MK2 inhibition restores radiosensitivity in *DDX21*‐deficient HNSCC cells. A–D. Colony formation assays of CAL27 and HN30 cells stably expressing vector control or *DDX21* knockdown, treated with or without the MK2 inhibitor MK2‐IN‐1 and irradiated with 0, 2, or 4 Gy. Colonies were quantified 21 days after irradiation. A and B show representative images, and C and D show the quantitative surviving fraction. E, F. Cell viability of CAL27 and HN30 cells with vector control or *DDX21* knockdown pretreated with or without MK2‐IN‐1, followed by irradiation at 0, 2, 4, or 6 Gy. Viability was assessed by CCK‐8 assay 3 d post‐irradiation. E shows CAL27 cells and F shows HN30 cells. G, H Representative neutral comet assay images and quantification of tail moments in CAL27 cells expressing shCtrl or sh‐DDX21 0, 4, 8 and 12 h after exposure to 10 Gy irradiation. I. Representative immunofluorescence images of γH2AX foci in vector control and *DDX21*‐knockdown cells treated with or without MK2‐IN‐1 at 0, 15 min, 1 h, 2 h, and 4 h after exposure to 10 Gy irradiation (scale bar = 5 µm). J. Fold change in the proportion of cells with >5 γH2AX foci relative to their respective non‐treated (NT) controls. K. Fold change in the proportion of cells with >5 γH2AX foci relative to their respective 0 h values after IR. L. Representative 53BP1 immunofluorescence images of CAL27 shCtrl and sh1 cells following 10 Gy irradiation. M,N. Proportions of cells containing more than five 53BP1 foci, expressed relative to the corresponding non‐irradiated control (M) or normalized to 0 h (N). Scale bars in I and L, 5 µm.

Given that p38‐mediated phosphorylation of MK2 at Thr334 is a critical regulator of the cellular response to DNA damage [29], we hypothesized that pharmacological inhibition of MK2 would increase the burden of IR‐induced DNA damage and restore radiosensitivity in DDX21‐knockdown cells. To test this hypothesis, stable *DDX21*‐knockdown cells were treated with the selective MK2 inhibitor (MK2‐IN‐1) [30, 31]. In vitro, *DDX21*‐knockdown cells displayed clear radioresistance, demonstrated by increased colony formation (Figure 5A–D) and elevated cell viability following IR (Figure 5E,F). Notably, MK2‐IN‐1 treatment effectively reversed this phenotype in *DDX21*‐knockdown cells, significantly reducing both colony numbers and viability. We next investigated the effects of DDX21 depletion on IR‐induced DNA damage and the associated DNA damage response. Neutral comet assays revealed lower tail moments in *DDX21*‐knockdown cells from 0 to 8 h after IR, consistent with a lower detectable DSB burden during the early post‐irradiation period (Figure 5G,H and Figure S7A,B). In parallel, DDX21 depletion reduced IR‐induced γH2AX and 53BP1 foci across multiple post‐irradiation time points, providing complementary evidence of an attenuated DNA damage response (Figure 5I–N and Figure S7C–E). Notably, MK2‐IN‐1 partially restored γH2AX accumulation in DDX21‐knockdown cells, further supporting the involvement of MK2 in the DDX21‐mediated DNA damage response. We additionally examined whether DDX21 depletion was accompanied by altered basal chromatin organization. Following MNase digestion, DDX21‐knockdown cells exhibited a reduced proportion of mononucleosomal DNA fragments and increased H3K9me3 levels (Figure S8). Collectively, these findings support a role for the DDX21–MK2 axis in regulating radiosensitivity through modulation of the DNA damage response and prompted us to further evaluate its function in vivo.

**FIGURE 6:**
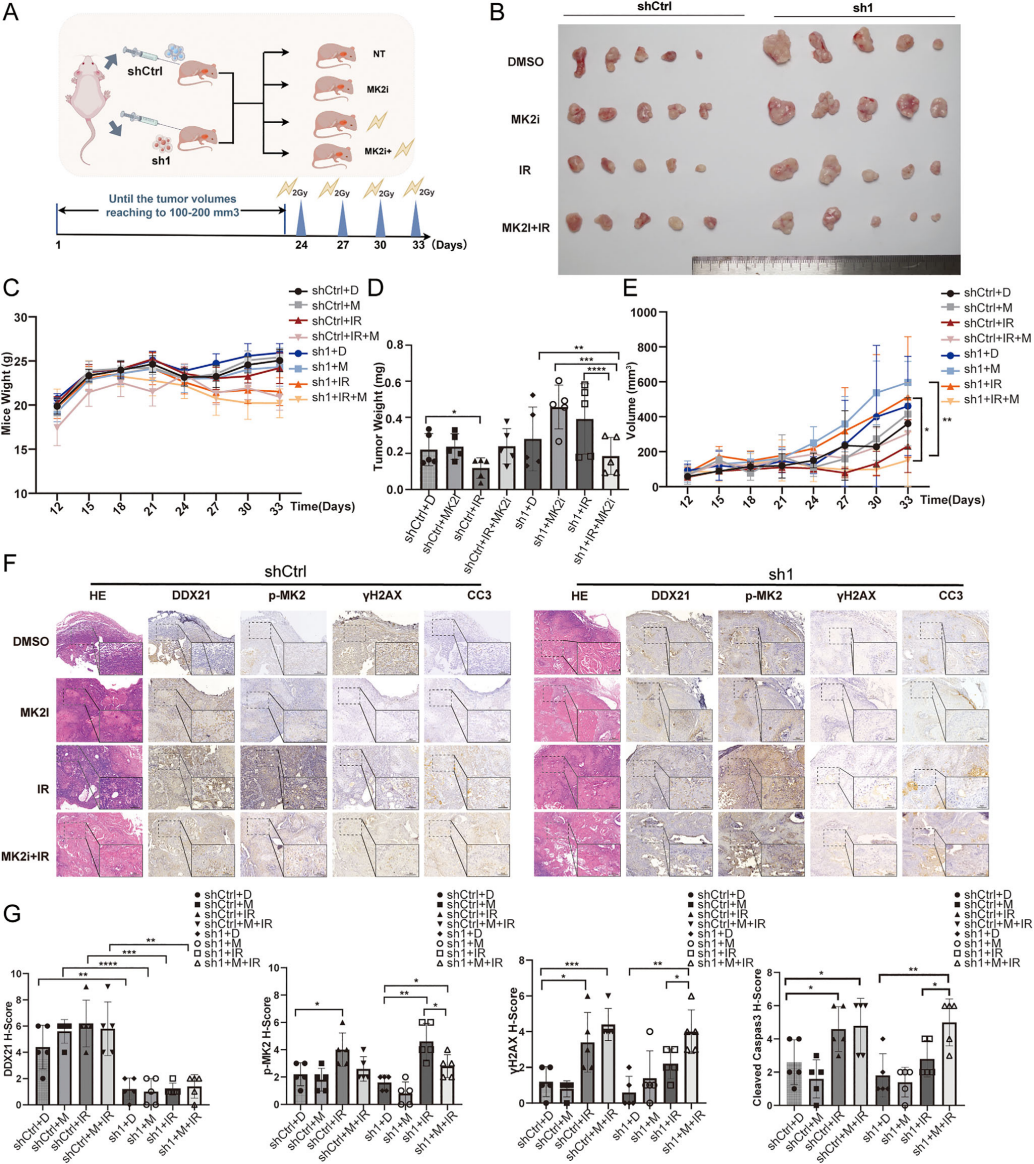
MK2 inhibition restores radiosensitivity in *DDX21*‐deficient HNSCC in vivo. In vivo studies: Subcutaneous xenografts were established using CAL27 cells expressing vector control or *DDX21* knockdown. Mice were randomized into four treatment groups: solvent control, MK2‐IN‐1 monotherapy, IR monotherapy, and combined IR + MK2‐IN‐1 therapy. A, B. Representative images of excised xenograft tumors from the four treatment groups. The figure was generated using Figdraw (www.figdraw.com). C. Weight changes in nude mice across different treatment groups (irradiation or MK2 inhibitor administration). D. Tumor weight comparison among groups at endpoint (*n* = 5; mean ± SD; *p* < 0.05). E. Tumor growth curves showing volume changes over time in each group. F. Representative immunohistochemical staining for DDX21, p‐MK2, γH2AX, and cleaved caspase‐3 in xenograft tumors (scale bar = 100 µm). G. Quantitative scoring of IHC staining for each target protein (*n* = 5; mean ± SD; *p* < 0.05).

To determine whether MK2 inhibition could similarly overcome radioresistance in vivo, we generated DDX21‐sh CAL27 xenograft models (Figure 6A,B). Compared with either monotherapy, combined IR and MK2‐IN‐1 treatment produced a significantly greater reduction in tumor weight (Figure 6D) and tumor volume (Figure 6E). Immunohistochemistry further demonstrated that combination therapy decreased p‐MK2 expression while increasing γH2AX and cleaved caspase‐3 levels (Figure 6F,G). Although a mild reduction in body weight was observed in the treatment groups, this effect is consistent with the anticipated physiological response to IR (Figure 6C). These in vitro and in vivo results indicate that the MK2 inhibitor MK2‐IN‐1 effectively restores radiosensitivity in *DDX21*‐deficient HNSCC by suppressing p‐MK2 and enhancing radiation‐induced DNA damage and apoptosis.

## 3 Discussion

Radiotherapy remains a central component of treatment for HNSCC, exerting its antitumor activity primarily through activation of the DNA damage response [32, 33, 34]. Nevertheless, overall survival outcomes for patients remain unsatisfactory [35]. Members of the DEAD‐box (DDX) RNA helicase family are increasingly recognized as key regulators of RNA metabolism and genomic stability [36]. Among them, DDX21 is a multifunctional helicase best known for its essential roles in rRNA biogenesis and transcriptional regulation [37], and it has also been implicated in maintaining genome integrity by coordinating replication stress responses and facilitating DNA repair [38]. In this study, we identify DDX21 as a favorable prognostic biomarker for postoperative radiotherapy, as elevated expression is associated with improved clinical outcomes. Mechanistically, irradiation enhances the interaction between DDX21 and MK2, resulting in suppression of MK2 kinase activity, accumulation of DNA damage, and increased apoptotic cell death. These findings highlight a previously unrecognized role for DDX21 in promoting radiosensitivity and establish it as a critical modulator of radiotherapy response in HNSCC.

Clinicopathological analysis revealed a significant association between DDX21 expression and tumor differentiation. Given the greater intrinsic radiosensitivity frequently observed in poorly differentiated tumors [39], DDX21 assessment may complement pathological grading and improve patient stratification for radiotherapy. In our cohort of patients who received postoperative radiotherapy, only 7.2% of tumors were p16‐positive, and p16 status did not differ significantly between the DDX21‐low and DDX21‐high groups. High DDX21 expression remained associated with better overall survival in the predominant p16‐negative subgroup. Moreover, DDX21 expression correlated positively with DDR scores in both HPV subgroups in GSE65858. These findings suggest that the associations of DDX21 with DDR activity and clinical outcome cannot be explained solely by HPV status and support its potential clinical relevance in HPV‐negative HNSCC, which constitutes a substantial patient population in Asia [40, 41]. Nevertheless, the small number of p16‐positive patients precluded subgroup‐specific prognostic analysis and formal testing of an interaction between DDX21 expression and HPV status. Consistent with previous studies linking DDX21 to genomic instability, we found that elevated DDX21 increased the radiation‐induced DSB burden and prolonged damage‐associated signaling in HNSCC cells. These effects were accompanied by a less compact chromatin state and increased apoptosis. Together, these findings suggest that DDX21 enhances radiosensitivity by augmenting the DNA damage response following irradiation.

The p38–MK2 pathway constitutes an important arm of the DNA damage response by coordinating cell‐cycle checkpoint signaling and cell survival following genotoxic stress [42, 43, 44]. MK2 phosphorylates Cdc25B and Cdc25C, thereby contributing to DNA damage‐induced cell‐cycle arrest [42]. Dependence on this pathway has been particularly well documented in p53‐deficient tumor cells, which rely on MK2‐mediated checkpoint signaling to survive genotoxic stress [43]. In HNSCC, IR‐induced MK2 activation has been associated with radioresistance, whereas elevated p‐MK2 levels correlate with poorer disease‐specific survival in patients with p16‐negative tumors [29]. In this study, we found that irradiation enhanced the interaction between DDX21 and MK2 and that DDX21 suppressed MK2 phosphorylation at Thr334 without detectably altering p38 phosphorylation. DDX21 increased the IR‐induced DNA damage burden and enhanced radiosensitivity, together with a shift toward a less compact chromatin state. Collectively, these findings identify the DDX21–MK2 axis as a previously unrecognized regulator of the DNA damage response and radiosensitivity following irradiation.

Given the essential cellular functions of RNA helicases, direct pharmacological targeting of these proteins remains challenging [45]. Building on our observation that *DDX21* depletion markedly reduces radiosensitivity, we propose an alternative strategy focused on modulating downstream effectors of DDX21. In this context, treatment with the MK2 inhibitor enhanced the effects of radiotherapy in *DDX21*‐deficient cells, highlighting the translational potential of targeting this pathway to overcome radioresistance. Despite the strength of these findings, several limitations should be acknowledged. First, our animal models were immunocompromised and therefore do not fully recapitulate the complex tumor immune microenvironment of clinical HNSCC. Moreover, the limited clinical sample size and cohort characteristics may restrict the generalizability and translational relevance of our findings. Finally, the development of highly selective MK2 inhibitors that specifically block Thr334 phosphorylation may further facilitate clinical translation and advance therapeutic opportunities.

## 4 Conclusion

In summary, we define a DDX21–MK2 signaling axis that governs DNA damage response regulation and radiosensitivity in HNSCC. Our findings identify a DDX21–MK2 regulatory axis in which DDX21 interacts with MK2, suppresses MK2 phosphorylation, and enhances the DNA damage response and radiosensitivity following irradiation. This work identifies the DDX21–MK2 interaction as a promising therapeutic target for radiosensitization and provides a mechanistic foundation for developing strategies to overcome radioresistance in HNSCC.

## 5 Experimental Methods

### 5.1 Cell Culture and Clinical Specimens

The CAL27, HN30, and 293T cell lines were provided by the Maxillofacial Tumor Research Center of Xiangya Hospital, Central South University. Cells were maintained in DMEM (Gibco, #8120506) supplemented with 10% fetal bovine serum (Biological Industries, #18224477) and cultured at 37°C in a humidified atmosphere containing 5% CO_2_. All cell lines were passaged fewer than 20 times and were routinely tested to confirm the absence of mycoplasma contamination. Cell line authentication was performed by Beijing Tsingke Biotech Co., Ltd.

For immunohistochemical analysis, 21 paired postoperative tumor and adjacent non‐tumor tissues from patients with HNSCC treated at the Department of Oral and Maxillofacial Surgery, the Second Xiangya Hospital of Central South University (January 2010 to December 2016) were collected. Tumor tissue specimens from 97 HNSCC patients who underwent postoperative radiotherapy were obtained from the Stomatology Center of Xiangya Hospital, Central South University (September 2019 to December 2022). Among the cohort, 90 patients were p16‐negative and 7 were p16‐positive, with 2 cases excluded due to missing survival‐related prognostic information. For tissue mRNA extraction, 14 paired tumor and adjacent non‐tumor tissues were collected from patients treated at Xiangya Hospital. The study was approved by the Ethics Committee of Xiangya Hospital (Ethics No. 202104814). Tumor specimens were sectioned, subjected to hematoxylin and eosin staining, and classified according to the NCCN (2023 edition) pathological criteria for head and neck tumors.

### 5.2 Bioinformatics Analysis

All datasets and gene lists used in this study were obtained from public databases. mRNA expression profiles and corresponding clinical information were retrieved from TCGA (TCGA‐HNSC) and the GEO databases (GSE13601, GSE25099 and GSE65858). A curated list of DDR‐related genes was downloaded from MSigDB v7.1 (Data S2), and members of the DDX family were compiled from previous publications [9]. Comprehensive bioinformatics analyses were conducted using HNSCC transcriptomic datasets to identify key regulators of the DNA damage response. The analytic workflow and selection criteria were as follows. First, differentially expressed genes between tumor and normal tissues in each dataset were identified using the Wilcoxon rank‐sum test. Genes with Benjamini–Hochberg (BH)‐adjusted *p* < 0.05 were considered differentially expressed. Second, gene set variation analysis (GSVA) was performed using the R package GSVA to calculate enrichment scores for DDR‐related pathways. Spearman correlation analysis was then conducted to evaluate associations between enrichment scores and gene expression levels. Genes with a correlation coefficient > 0.45 and *p* < 0.05 were selected for further analysis. In addition, patients in each cohort were stratified into DDR‐high and DDR‐low groups (DDR high and DDR low) based on the median enrichment score. Differentially expressed genes between these two groups were identified using the Wilcoxon rank‐sum test with BH correction.

### 5.3 Plasmid Construction and Cell Transfection

Overexpression plasmids (pLVX‐SBP‐FLAG‐DDX21 and pLVX‐SBP‐FLAG‐vector) and knockdown constructs (Tet‐on‐ctrl, Tet‐on‐DDX21‐sh1, Tet‐on‐DDX21‐sh2) were purchased from Beijing Qingke Biotechnology Co., Ltd. CAL27 and HN30 cells were transfected at optimal density using Lipofectamine 3000 (Invitrogen, L3000015) according to the manufacturer's protocol (Figure S1B). For Tet‐on‐mediated shRNA induction, cells were treated with doxycycline (0.2 µg mL^−1^) for 72 h before each experiment (Figure S1C,D). Lentiviral packaging was performed using psPAX2 and pMD2.G plasmids provided by the Key Laboratory of Molecular Radiation Oncology, Hunan Province.

### 5.4 Immunofluorescence

CAL27 control cells and DDX21‐stably overexpressing cells were seeded onto 14‐mm coverslips and subsequently exposed to 10 Gy ionizing radiation (IR), and harvested at designated time points. Cells were fixed with 4% paraformaldehyde (PFA) for 15 min at room temperature and permeabilized with 0.2% Triton X‐100 in PBS for 10 min. After blocking with 2% BSA for 30 min, cells were incubated overnight at 4°C with primary antibodies against DDX21 (Abcam, ab182156; 1:400), γH2AX (Millipore, 05‐636;1:1000), and Flag (Sigma, F1804; 1:500). Following three washes with PBST, cells were incubated with Alexa Fluor 488–conjugated goat anti‐rabbit IgG (Jackson, 111‐545‐003; 1:800) for 1 h at room temperature in the dark. Nuclei were counterstained with DAPI, and coverslips were mounted with antifade mounting medium (Boster, AR1109). Images were captured using a Leica TCS SP8 confocal microscope.

### 5.5 Xenograft Experiments

All animal procedures were approved by the Animal Ethics Committee of Xiangya Hospital, Central South University (Ethics No.202104814). For DDX21 overexpression studies, 5 × 10^6^ CAL27 control cells and 5 × 10^6^ DDX21‐overexpressing cells were each suspended in 50 µL Matrigel and injected subcutaneously into 4–6‐week‐old nude mice. When tumors reached 100–200 mm^3^ [46, 47], mice were randomized by cell type and treated with 2 Gy irradiation every three days for a total of three doses using a PRECISION X‐RAY SmART+ system (USA, Precision X‐ray Inc.). For knockdown experiments, 5 × 10^6^ CAL27 control or DDX21‐silenced cells were implanted using the same protocol. Once tumors reached 50–60 mm^3^, mice were allocated to four groups: MK2‐IN‐1 treatment (70 µL of 0.5 µg µL^−1^ every other day), irradiation (2 Gy every three days), combined therapy, or control. Tumor volume and body weight were monitored daily. Tumor volume (mm^3^) was calculated as 0.5 × length × width^2^.

### 5.6 Cell Counting Kit‐8 (CCK‐8) Assay

Cell viability after irradiation was assessed using the CCK‐8 assay (MedChemExpress, HY‐K0301). CAL27 and HN30 cells in control, DDX21 overexpression, or knockdown groups were exposed to graded doses of IR. After treatment, culture medium was replaced with serum‐free medium containing CCK‐8 reagent, and cells were incubated for 1 h in the dark. Absorbance was measured at 450 nm.

### 5.7 Western Blotting

Total protein was extracted from HNSCC cell lines and quantified prior to analysis. Equal amounts of protein were resolved by 10% or 12.5% SDS‐PAGE (Epizyme Biotech, PG112, PG213) and transferred onto PVDF membranes (Millipore, USA). Membranes were blocked with 5% nonfat milk for 20 min at room temperature and incubated overnight at 4°C with primary antibodies against DDX21, MK2(MAPKAPK2), phospho‐MAPKAPK2 (Thr334), p38, phospho‐P38, Flag, β‐tubulin, cleaved caspase‐3, caspase‐3, 53BP1 and γH2AX. Membranes were then incubated with HRP‐conjugated secondary antibodies (Boster, BA1050, BA1054, 1:5000) for 1 h at room temperature. Signals were detected using enhanced chemiluminescence and visualized with a Bio‐Rad imaging system.

### 5.8 RT‐qPCR

Total RNA was extracted from 14 paired tumor and adjacent non‐tumor tissues and reverse‐transcribed into cDNA. Quantitative real‐time PCR was performed using SYBR Green chemistry to determine DDX21 mRNA expression. Thermal cycling conditions were applied as previously described. Primer sequences were as follows (5′→3′): DDX21 forward: GAGGAGCCATCTCAAAATGACA; DDX21 reverse: GGGTTACAGTCCGGTTCAGG. GAPDH forward: GGAGCGAGATCCCTCCAAAAT; GAPDH reverse: GGCTGTTGTCATACTTCTCATGG. Each reaction was performed in triplicate.

### 5.9 Neutral Comet Assay

CAL27 cells stably overexpressing DDX21 or transfected with empty vector were exposed to 10 Gy of IR. After irradiation, cells and supernatant were collected, centrifuged at 2,500 rpm, washed with ice‐cold PBS, and resuspended at a density of 3 × 10^5^ cells mL^−1^. The cell suspension was mixed with low‐melting point agarose at 37°C (1:10) and 50 µL of the mixture was applied to CometSlides, evenly spread, and allowed to solidify at 4°C for 30 min in the dark. Slides were lysed overnight at 4°C and then equilibrated in precooled neutral electrophoresis buffer for 30 min. Electrophoresis was performed at 17 V for 40 min at 4°C in 450 mL of buffer, with the liquid level maintained ≤ 0.5 cm above the slide surface. Slides were subsequently incubated in DNA precipitation solution for 30 min at room temperature, followed by immersion in 70% ethanol for another 30 min. The slides were dried at 37°C for 10–15 min, stained in the dark with diluted SYBR Green I (100 µL per slide) for 30 min, and air‐dried. DNA tail lengths were visualized and quantified using a fluorescence microscope.

### 5.10 Colony Formation Assay

DDX21‐overexpressing, DDX21‐knockdown, and corresponding control cells were seeded into six‐well plates at a density of 600 cells per well, with three replicates per group, in 2 mL complete culture medium (DMEM supplemented with 10% FBS). Following exposure to graded doses of irradiation, cells were cultured at 37°C for 21 d, with media refreshed every 5 d. After colony formation, cells were rinsed twice with PBS, fixed with 1 mL of 4% paraformaldehyde for 20 min, washed again, and stained with 1 mL of 1% crystal violet for 30 min. Plates were subsequently rinsed, air‐dried, photographed, and colonies were quantified.

### 5.11 Apoptosis Assay

CAL27 and HN30 cells stably overexpressing DDX21 or transfected with empty vector were irradiated with 0 or 10 Gy. Both floating and adherent cells were collected. Adherent cells were gently detached using trypsin without EDTA, combined with non‐adherent cells, centrifuged at 1000 × *g* for 5 min, washed, and resuspended in PBS. A total of 5–10 × 10^5^ cells were incubated with 190 µL annexin V‐FITC binding buffer and 10 µL annexin V‐FITC for 10 min at room temperature in the dark. After washing and centrifugation, samples were resuspended in 195 µL binding buffer and stained with 5 µL propidium iodide (PI) before immediate flow cytometric analysis. Apoptosis was analyzed immediately by flow cytometry using 488 nm excitation, with Annexin V‐FITC detected in the FL1 channel and PI in the FL3 channel.

### 5.12 Sister Chromatid Exchange (SCE) Assay

CAL27 cells were seeded in 10‐cm culture dishes at 10–15% confluence. After cell attachment, BrdU was added to a final concentration of 20 µm, and cells were cultured in the dark for two cell cycles. Four hours before harvesting, colchicine (0.1–0.2 µg mL^−1^) was added. Cells were then collected, centrifuged at 500 × *g* for 10 min, and treated with 75 mm KCl at 37°C for 30 min. Cells were fixed with methanol–acetic acid solution, washed repeatedly, and dropped onto humidified slides. After air‐drying, slides were incubated in 2× SSC at 55°C and exposed to ultraviolet light (99.9 J) for 21 min, followed by rinsing, drying, and staining with 30% Giemsa for 2 min. After a final rinse and drying, SCE events were examined microscopically.

### 5.13 Co‐Immunoprecipitation Combined With Mass Spectrometry (Co‐IP/MS)

Neutralization buffer consisted of 50 mm Tris–HCl (pH 8.0), 150 mm NaCl, and 1 mm EDTA. Washing buffer contained 20 mm Tris–HCl (pH 8.0), 150 mm NaCl, 1 mm EDTA, and 0.2% Triton X‐100. When CAL27 cells reached approximately 50% confluence, Flag‐tagged DDX21 plasmid or empty vector was transfected, and cells were cultured for 24 h. Cells were washed three times with PBS, lysed in 300 µL lysis buffer on ice for 10 min, mixed with an equal volume of neutralization buffer, and incubated on ice for an additional 20 min. Lysates were centrifuged at 12 000 rpm for 10 min at 4°C, and the supernatant was incubated with anti‐Flag M2 agarose beads overnight at 4°C with rotation. The beads were washed five times with IP buffer. Immunoprecipitated proteins were analyzed by immunoblotting. For mass spectrometry, a 2‐cm gel slice containing the target protein was excised and analyzed using an Orbitrap Exploris 480 system (Germany). Protein classification was performed using the PANTHER database, and functional enrichment was analyzed. KEGG pathway analysis was conducted using the R/Bioconductor package clusterProfiler (version 3.16.1).

### 5.14 In Vitro Kinase Assay

293T cells were transfected with Flag‐DDX21 plasmid, and cells in the irradiation group were exposed to 10 Gy X‐rays 48 h post‐transfection. Flag‐DDX21 protein was purified by co‐immunoprecipitation and eluted with Flag peptide. Recombinant p38 protein was expressed in IPTG‐induced *E. coli* BL21 (DE3), harvested, resuspended, and lysed in IP buffer containing protease inhibitors. Cells were sonicated at 20% power for 9 s, followed by 1‐minute intervals on ice, repeated three times, and centrifuged at 12 000 × *g* for 10 min at 4°C. The supernatant containing p38 was collected. Commercial GST‐MK2 protein (Sino Biological, China) was used as the substrate. For kinase reactions, 20 µL GST magnetic beads were added to mixtures containing Flag‐DDX21, p38, and GST‐MK2, and incubated at 4°C for 1–2 h. After washing three times, ATP (10 mm; Cell Signaling Technology, 9804S) and 10× kinase buffer (Cell Signaling Technology, 9802S) were added. Reactions were carried out at 32°C for 45 min and terminated by adding SDS loading buffer for Western blot analysis.

### 5.15 Histological and Immunohistochemical Analysis

For immunohistochemical staining, 4‐µm‐thick sections of normal and tumor tissues were deparaffinized, rehydrated, and subjected to antigen retrieval in sodium citrate buffer (pH 6.0). Endogenous peroxidase activity was quenched with 3% hydrogen peroxide, and nonspecific binding was blocked with bovine serum albumin (BSA). The sections were then incubated with the primary antibodies overnight at 4°C. The sections were subsequently incubated with the appropriate secondary antibodies, and immunoreactivity was visualized using 0.03% 3,3′‐diaminobenzidine (DAB). Immunohistochemical staining was independently evaluated by two pathologists who were blinded to the clinicopathological characteristics and clinical outcomes of the patients. Each specimen was scored according to staining intensity and the proportion of positively stained tumor cells. Staining intensity was scored as 0 (absent), 1 (weak), 2 (moderate), or 3 (strong). The proportion of positively stained tumor cells was scored as 1 (0–25%), 2 (>25–50%), 3 (>50–75%), or 4 (>75–100%). The final immunoreactive score (IRS) was calculated by multiplying the staining‐intensity score by the positive‐cell proportion score, yielding a total score ranging from 0 to 12. Discrepant assessments were jointly reviewed by the two pathologists until consensus was reached. The median IRS of the cohort was used as the cutoff for expression stratification. Patients with IRS values equal to or below the median were classified as the DDX21‐low group, whereas those with scores above the median were classified as the DDX21‐high group [48].

### 5.16 Micrococcal Nuclease Digestion

Cells were washed once with ice‐cold phosphate‐buffered saline (PBS) and harvested in 2 mL of ice‐cold PBS supplemented with a protease inhibitor cocktail. After centrifugation at 900 × *g* for 5 min at 4°C, the cell pellet was gently resuspended in 50 µL of ice‐cold RBS buffer containing 10 mm Tris‐HCl (pH 7.4), 10 mm NaCl, and 3 mm CaCl_2_. Subsequently, 1.3 mL of RBS buffer containing 0.1% NP‐40 was added to induce hypotonic cell lysis. Nuclei were collected by centrifugation at 500 × *g* for 10 min at 4°C, resuspended in RBS buffer, and counted. Equal numbers of nuclei isolated from CAL27 and HN30 cells were digested with micrococcal nuclease (MNase; final concentration, 0.03 U µL^−^^1^) in a final reaction volume of 20 µL. Digestion was performed at 37°C for 15 min and terminated by adding EDTA to a final concentration of 40 mm. The samples were then treated with RNase A at a final concentration of 100 µg mL^−^^1^ for 30 min at 37°C, followed by incubation with proteinase K at a final concentration of 1.2 µg µL^−^^1^ for 1 h at 45°C. Genomic DNA was purified by phenol–chloroform extraction, and 200 ng of the purified DNA was separated by electrophoresis on a 2% agarose gel.

### 5.17 Statistical Analysis

All statistical analyses were conducted using SPSS version 26.0 (IBM, Armonk, NY, USA). Data are presented as mean ± standard deviation (SD). Differences between two groups were evaluated using an independent‐samples t‐test, while comparisons among multiple groups were performed by one‐way analysis of variance (ANOVA). The chi‐square test was applied for analysis of categorical variables, and Fisher’s exact test was used when expected cell counts were < 5. Kaplan–Meier survival curves were compared using the log‐rank test. A two‐tailed *p* < 0.05 was considered statistically significant.

## Author Contributions

**Tianru Yang**: methodology, data curation, software, formal analysis, writing – original draft, investigation, project administration, resources. **Ting Shen**: writing – review and editing, methodology, funding acquisition, conceptualization. **Yanfang Qiu**: methodology, data curation, funding acquisition. **Wei Zhang**: visualization, formal analysis, methodology, software. **Ziran Zheng**: methodology, visualization. **Jiayi Sun**: writing – review and editing. **Yuedi Yao**: conceptualization, validation. **Mianfeng Yao**: methodology, data curation, formal analysis. **Jiang Li**: conceptualization, methodology, data curation, formal analysis, funding acquisition, writing – review and editing, supervision.

## Conflicts of Interest

The authors declare no conflicts of interest.

## Supporting information




**Supporting File 1**:: advs77747‐sup‐0001‐SuppMat.docx.

**Supporting File 2**: advs77747‐sup‐0002‐SuppMat.xlsx.

## Data Availability

The public datasets including TCGA‑HNSC, GSE13601, GSE25099 and GSE65858 analyzed in this study are openly available from the TCGA GDC portal (https://portal.gdc.cancer.gov/) and the NCBI Gene Expression Omnibus (GEO) database (https://www.ncbi.nlm.nih.gov/geo/), respectively. The anonymized clinical and IHC datasets derived from our in‑house HNSCC patient cohort cannot be publicly released due to institutional ethical restrictions and patient privacy protection. Qualified researchers may request the de‑identified raw data by contacting the corresponding author upon reasonable request.

## References

[1] Y. Razvi , S. Chan , L. Zhang , et al., “A Review of the Rapid Response Radiotherapy Program in Patients with Advanced Cancer Referred for Palliative Radiotherapy over Two Decades,” Supportive Care in Cancer 27 (2019): 2131–2134, 10.1007/s00520-018-4474-9. 30246224

[2] J. P. Machiels , C. René Leemans , W. Golusinski , C. Grau , L. Licitra , and V. Gregoire , “Reprint of “Squamous Cell Carcinoma of the Oral Cavity, Larynx, Oropharynx and Hypopharynx: EHNS‐ESMO‐ESTRO Clinical Practice Guidelines for Diagnosis, Treatment and Follow‐Up”,” Oral Oncology 113 (2021): 105042, 10.1016/j.oraloncology.2020.105042. 33583513

[3] L. Q. M. Chow , “Head and Neck Cancer,” New England Journal of Medicine 382, no. 1 (2020): 60–72, 10.1056/NEJMra1715715. 31893516

[4] Y. Gong , L. Bao , T. Xu , et al., “The Tumor Ecosystem in Head and Neck Squamous Cell Carcinoma and Advances in Ecotherapy,” Molecular Cancer 22, no. 1 (2023): 68, 10.1186/s12943-023-01769-z. 37024932 PMC10077663

[5] K. M. Burcher , A. T. Faucheux , J. W. Lantz , et al., “Prevalence of DNA Repair Gene Mutations in Blood and Tumor Tissue and Impact on Prognosis and Treatment in HNSCC,” Cancers 13, no. 13 (2021): 3118, 10.3390/cancers13133118. 34206538 PMC8267691

[6] P. Sanese , K. De Marco , M. Lepore Signorile , et al., “The Novel SMYD3 Inhibitor EM127 Impairs DNA Repair Response to Chemotherapy‐Induced DNA Damage and Reverses Cancer Chemoresistance,” Journal of Experimental & Clinical Cancer Research 43, no. 1 (2024): 151, 10.1186/s13046-024-03078-9. 38812026 PMC11137994

[7] H. L. Wilson , R. B. D'Agostino Jr , N. Meegalla , R. Petro , et al., “The Prognostic and Therapeutic Value of the Mutational Profile of Blood and Tumor Tissue in Head and Neck Squamous Cell Carcinoma,” The Oncologist 26, no. 2 (2021): e279–e289, 10.1002/onco.13573. 33098199 PMC7873320

[8] P. B. M. Essers , M. van der Heijden , C. V. M. Verhagen , et al., “Drug Sensitivity Prediction Models Reveal a Link between DNA Repair Defects and Poor Prognosis in HNSCC,” Cancer Research 79, no. 21 (2019): 5597–5611, 10.1158/0008-5472.Can-18-3388. 31515237

[9] J. Xie , M. Wen , J. Zhang , et al., “The Roles of RNA Helicases in DNA Damage Repair and Tumorigenesis Reveal Precision Therapeutic Strategies,” *Cancer Research* 82, no. 5 (2022): 872–884, 10.1158/0008-5472.CAN-21-2187. 34987058

[10] W. Zhang , W. Liu , L. Jia , et al., “Targeting KDM4A Epigenetically Activates Tumor‐Cell‐Intrinsic Immunity by Inducing DNA Replication Stress,” Molecular Cell 81, no. 10 (2021): 2148–2165.e9, 10.1016/j.molcel.2021.02.038. 33743195 PMC8141018

[11] S. Movilla , M. Roca , V. Moliner , and A. Magistrato , “Molecular Basis of RNA‐Driven ATP Hydrolysis in DExH‐Box Helicases,” Journal of the American Chemical Society 145, no. 12 (2023): 6691–6701, 10.1021/jacs.2c11980. 36926902

[12] K. E. Bohnsack , S. Yi , S. Venus , E. Jankowsky , and M. T. Bohnsack , “Cellular Functions of Eukaryotic RNA Helicases and Their Links to human Diseases,” Nature Reviews Molecular Cell Biology 24, no. 10 (2023): 749–769, 10.1038/s41580-023-00628-5. 37474727

[13] J. Castro , M. H. Daniels , D. Brennan , et al., “A Potent, Selective, Small‐Molecule Inhibitor of DHX9 Abrogates Proliferation of Microsatellite Instable Cancers with Deficient Mismatch Repair,” Cancer Research 85 (2025): 758–776, 10.1158/0008-5472.Can-24-0397. 39589774 PMC11831107

[14] H. Fu , M. Huang , H. Wu , et al., “SART3 promotes Homologous Recombination Repair by Stimulating DNA‐RNA Hybrids Removal and DNA End Resection,” Nature Communications 16 (2025): 2244, 10.1038/s41467-025-57599-8. PMC11885473 40050279

[15] Y. Xiao , J. Fan , Z. Li , and Y. Hou , “DDX21 at the Nexus of RNA Metabolism, Cancer Oncogenesis, and Host–Virus Crosstalk: Decoding Its Biomarker Potential and Therapeutic Implications,” International Journal of Molecular Sciences 25, no. 24 (2024): 13581, 10.3390/ijms252413581. 39769343 PMC11676383

[16] H. Gao , H. Wei , Y. Yang , et al., “Phase Separation of DDX21 Promotes Colorectal Cancer Metastasis via MCM5‐Dependent EMT Pathway,” Oncogene 42, no. 21 (2023): 1704–1715, 10.1038/s41388-023-02687-6. 37029300 PMC10202810

[17] K. Su , Z. Zhao , Y. Wang , et al., “NAT10 resolves Harmful Nucleolar R‐Loops Depending on Its Helicase Domain and Acetylation of DDX21,” Cell Communication and Signaling 22, no. 1 (2024): 490, 10.1186/s12964-024-01869-3. 39394182 PMC11468200

[18] G. Zhang , R. Yang , B. Wang , et al., “TRIP13 regulates Progression of Gastric Cancer through Stabilising the Expression of DDX21,” Cell Death & Disease 15, no. 8 (2024): 622, 10.1038/s41419-024-07012-x. 39187490 PMC11347623

[19] R. Huang , Z. Yang , Q. Liu , B. Liu , X. Ding , and Z. Wang , “CircRNA DDX21 Acts as a Prognostic Factor and Sponge of miR‐1264/QKI Axis to Weaken the Progression of Triple‐Negative Breast Cancer,” Clinical and Translational Medicine 12, no. 5 (2022): 768, 10.1002/ctm2.768. PMC9076009 35522944

[20] M. S. Aggerbeck , Gene Profile for Chemotherapy Response in Head and Neck Carcinoma, in NCBI Gene Expression Omnibus (GEO),(2013).

[21] S. Shimizu , Gene Expression Profiles of Head and Neck Squamous Cell Carcinoma (HNSCC), in NCBI Gene Expression Omnibus (GEO), (2007).

[22] G.D.C. (GDC) , TCGA Head and Neck Squamous Cell Carcinoma (TCGA‐HNSC) Dataset, the Cancer Genome Atlas (TCGA) (2023).

[23] G. Wichmann , M. Rosolowski , K. Krohn , et al., “The Role of HPV RNA Transcription, Immune Response‐Related Gene Expression and Disruptive TP53 Mutations in Diagnostic and Prognostic Profiling of Head and Neck Cancer,” International Journal of Cancer 137, no. 12 (2015): 2846–2857, 10.1002/ijc.29649. 26095926

[24] G. Wichmann and M. Rosolowski , Gene Expression Patterns and TP53 Mutations are Associated with HPV RNA Status, Lymph Node Metastasis, and Survival in Head And Neck Cancer, in NCBI Gene Expression Omnibus, (2015).

[25] H. Salim , N. S. Akbar , D. Zong , et al., “miRNA‐214 Modulates Radiotherapy Response of Non‐Small Cell Lung Cancer Cells through Regulation of p38MAPK, Apoptosis and Senescence,” British Journal of Cancer 107, no. 8 (2012): 1361–1373, 10.1038/bjc.2012.382. 22929890 PMC3494421

[26] M. J. Kim , S. Y. Choi , I. C. Park , et al., “Opposing Roles of c‐Jun NH2‐Terminal Kinase and p38 Mitogen‐Activated Protein Kinase in the Cellular Response to Ionizing Radiation in Human Cervical Cancer Cells,” Molecular Cancer Research 6, no. 11 (2008): 1718–1731, 10.1158/1541-7786.Mcr-08-003. 19010820

[27] N. García‐Flores , J. Jiménez‐Suárez , C. Garnés‐García , et al., “P38 MAPK and Radiotherapy: Foes or Friends?,” Cancers (Basel) 15 (2023): 861, 10.3390/cancers15030861. 36765819 PMC9913882

[28] P. M. Grierson , P. B. Dodhiawala , Y. Cheng , et al., “The MK2/Hsp27 Axis Is a Major Survival Mechanism for Pancreatic Ductal Adenocarcinoma under Genotoxic Stress,” Science Translational Medicine 13 (2021): abb5445, 10.1126/scitranslmed.abb5445. PMC9475471 34851698

[29] K. L. Berggren , S. R. Cruz , M. D. Hixon , et al., “MAPKAPK2 (MK2) inhibition Mediates Radiation‐Induced Inflammatory Cytokine Production and Tumor Growth in Head and Neck Squamous Cell Carcinoma,” Oncogene 38, no. 48 (2019): 7329–7341, 10.1038/s41388-019-0945-9. 31417185 PMC6883149

[30] S. Penninckx , E. Pariset , E. Cekanaviciute , and S. V. Costes , “Quantification of Radiation‐Induced DNA Double Strand Break Repair Foci to Evaluate and Predict Biological Responses to Ionizing Radiation,” NAR Cancer 3, no. 4 (2021): zcab046, 10.1093/narcan/zcab046. 35692378 PMC8693576

[31] Y. Zhang , H. Ding , X. Wang , et al., “MK2 promotes Tfcp2l1 Degradation via β‐TrCP Ubiquitin Ligase to Regulate Mouse Embryonic Stem Cell Self‐Renewal,” Cell Reports 37, no. 5 (2021): 109949, 10.1016/j.celrep.2021.109949. 34731635

[32] R. M. Chabanon , M. Rouanne , C. J. Lord , J. C. Soria , P. Pasero , and S. Postel‐Vinay , “Targeting the DNA Damage Response in Immuno‐Oncology: Developments and Opportunities,” Nature Reviews Cancer 21, no. 11 (2021): 701–717, 10.1038/s41568-021-00386-6. 34376827

[33] R. X. Huang and P. K. Zhou , “DNA Damage Response Signaling Pathways and Targets for Radiotherapy Sensitization in Cancer,” Signal Transduction and Targeted Therapy 5 (2020): 60, 10.1038/s41392-020-0150-x. 32355263 PMC7192953

[34] S. J. Bright , M. Manandhar , D. B. Flint , et al., “ATR Inhibition Radiosensitizes Cells through Augmented DNA Damage and G2 Cell Cycle Arrest Abrogation,” JCI Insight 9, no. 19 (2024): e179599, 10.1172/jci.insight.179599. 39235982 PMC11466186

[35] L. K. Mell , P. A. Torres‐Saavedra , S. J. Wong , et al., “Radiotherapy with cetuximab or durvalumab for Locoregionally Advanced Head and Neck Cancer in Patients with a Contraindication to Cisplatin (NRG‐HN004): an Open‐Label, Multicentre, Parallel‐Group, Randomised, Phase 2/3 Trial,” The Lancet Oncology 25, no. 12 (2024): 1576–1588, 10.1016/s1470-2045(24)00507-2. 39551064 PMC11726348

[36] X. Ma , T. Lu , Y. Yang , et al., “DEAD‐Box Helicase family Proteins: Emerging Targets in Digestive System Cancers and Advances in Targeted Drug Development,” Journal of Translational Medicine 22, no. 1 (2024): 1120, 10.1186/s12967-024-05930-0. 39707322 PMC11662784

[37] K. Koltowska , K. S. Okuda , M. Gloger , et al., “The RNA Helicase Ddx21 Controls Vegfc‐Driven Developmental Lymphangiogenesis by Balancing Endothelial Cell Ribosome Biogenesis and p53 Function,” Nature Cell Biology 23, no. 11 (2021): 1136–1147, 10.1038/s41556-021-00784-w. 34750583

[38] J. D. Hao , Q. L. Liu , M. X. Liu , et al., “DDX21 mediates co‐Transcriptional RNA m6A Modification to Promote Transcription Termination and Genome Stability,” Molecular Cell 84, no. 9 (2024): 1711–1726.e11, 10.1016/j.molcel.2024.03.006. 38569554

[39] L. Fan , Z. Yang , M. Chang , Z. Chen , and Q. Wen , “CT‐Based Delta‐Radiomics Nomogram to Predict Pathological Complete Response after Neoadjuvant Chemoradiotherapy in Esophageal Squamous Cell Carcinoma Patients,” Journal of Translational Medicine 22, no. 1 (2024): 579, 10.1186/s12967-024-05392-4. 38890720 PMC11186275

[40] L. Guo , F. Yang , Y. Yin , et al., “Prevalence of Human Papillomavirus Type‐16 in Head and Neck Cancer among the Chinese Population: A Meta‐Analysis,” Frontiers in Oncology 8 (2018): 619, 10.3389/fonc.2018.00619. 30619756 PMC6299118

[41] X. Castellsagué , L. Alemany , M. Quer , et al., “HPV Involvement in Head and Neck Cancers: Comprehensive Assessment of Biomarkers in 3680 Patients,” JNCI: Journal of the National Cancer Institute 108 (2016): djv403, 10.1093/jnci/djv403. 26823521

[42] I. A. Manke , A. Nguyen , D. Lim , M. Q. Stewart , A. E. Elia , and M. B. Yaffe , “MAPKAP Kinase‐2 Is a Cell Cycle Checkpoint Kinase That Regulates the G2/M Transition and S Phase Progression in Response to UV Irradiation,” Molecular Cell 17, no. 1 (2005): 37–48, 10.1016/j.molcel.2004.11.021. 15629715

[43] H. C. Reinhardt , A. S. Aslanian , J. A. Lees , and M. B. Yaffe , “p53‐Deficient Cells Rely on ATM‐ and ATR‐Mediated Checkpoint Signaling through the p38MAPK/MK2 Pathway for Survival after DNA Damage,” Cancer Cell 11, no. 2 (2007): 175–189, 10.1016/j.ccr.2006.11.024. 17292828 PMC2742175

[44] H. C. Reinhardt , P. Hasskamp , I. Schmedding , et al., “DNA Damage Activates a Spatially Distinct Late Cytoplasmic Cell‐Cycle Checkpoint Network Controlled by MK2‐Mediated RNA Stabilization,” Molecular Cell 40, no. 1 (2010): 34–49, 10.1016/j.molcel.2010.09.018. 20932473 PMC3030122

[45] O. Andrisani , Q. Liu , P. Kehn , and M. Gale Jr , “Biological Functions of DEAD/DEAH‐Box RNA Helicases in Health and Disease,” Nature Immunology 23, no. 3 (2022): 354–357, 10.1038/s41590-022-01149-7. 35194205 PMC10259094

[46] S. Corrales‐Guerrero , T. Cui , V. Castro‐Aceituno , et al., “Inhibition of RRM2 Radiosensitizes Glioblastoma and Uncovers Synthetic Lethality in Combination with Targeting CHK1,” Cancer Letters 570 (2023): 216308, 10.1016/j.canlet.2023.216308. 37482342

[47] F. T. Zenke , A. Zimmermann , C. Sirrenberg , et al., “Pharmacologic Inhibitor of DNA‐PK, M3814, Potentiates Radiotherapy and Regresses Human Tumors in Mouse Models,” Molecular Cancer Therapeutics 19, no. 5 (2020): 1091–1101, 10.1158/1535-7163.Mct-19-0734. 32220971

[48] W. Remmele and H. E. Stegner , “Recommendation for Uniform Definition of an Immunoreactive Score (IRS) for Immunohistochemical Estrogen Receptor Detection (ER‐ICA) in Breast Cancer Tissue,” Der Pathologe 8 (1987): 138–140. 3303008

