# Supplementary material for: DDX21 Enhances Radiosensitivity in Head and Neck Squamous Cell Carcinoma by Suppressing MK2‐Mediated DNA Damage Response
Source: Adv Sci (Weinh). 2026 Sep 16:e77747. Online ahead of print. doi: 10.1002/advs.77747 (PMC13583123; doi:10.1002/advs.77747)
Supplement: Supplementary file 1 [file ADVS-9999-e77747-s002.docx]

Supporting Information 1

Title：DDX21 Enhances Radiosensitivity in Head and Neck Squamous Cell Carcinoma by Suppressing MK2-mediated DNA Damage Response

Tianru Yang^1,2,3^, Ting Shen^4,5^, Yanfang Qiu^6^, Wei Zhang^3,7^, Ziran Zheng^4,5^, Jiayi Sun^1^, Yuedi Yao^1^, Mianfeng Yao^8*^, Jiang Li^1 ,3*^

**Figure S1. Validation of DDX21 overexpression and inducible knockdown in HNSCC cells.** A) Basal DDX21 protein expression in seven HNSCC cell lines; CAL27 and HN30 cells were selected for subsequent experiments. B) Immunoblot and densitometric analyses of DDX21 in control (Ctrl) and DDX21-overexpressing (OE) CAL27 and HN30 cells. C) DDX21 expression following treatment with the indicated doxycycline (DOX) concentrations in cells expressing control shRNA (shCtrl) or two independent DDX21-targeting shRNAs (sh1 and sh2); 0.2 μg mL⁻¹ DOX was used subsequently. D) Immunoblot and densitometric analyses of DOX-inducible DDX21 knockdown. DDX21 levels were normalized to the corresponding loading controls. Data are presented as mean ± SD, *n* = 3 independent biological experiments. Statistical significance was determined using two-tailed unpaired Student’s *t*-tests in B and D. **P* < 0.05, ***P* < 0.01; ns, not significant.

**Figure S2. DDX21 expression in HNSCC tissues and its effects on cell proliferation.**

A) Representative H&E and DDX21 immunohistochemical staining of paired HNSCC tumor (CA) and adjacent non-tumor (PCA) tissues. B) DDX21 IHC scores in paired CA and PCA tissues (*n* = 21). C) RT-qPCR analysis of DDX21 mRNA expression in paired CA and PCA tissues (*n* = 14). D,F) CCK-8 assays of control (Ctrl) and DDX21-overexpressing (OE) CAL27 and HN30 cells, respectively. E,G) Representative colony-formation assays and corresponding quantification in Ctrl and DDX21-OE CAL27 and HN30 cells, respectively. H) Violin plots comparing the expression of selected DNA damage response (DDR)-related genes between HPV-positive and HPV-negative tumors in the GSE65858 cohort. I) Violin plots comparing DDX21 mRNA expression between HPV-positive and HPV-negative tumors in the GSE65858 cohort. J,K) Correlations between DDX21 expression and DNA damage response scores in the indicated HPV-stratified cohorts. Lines and shaded areas indicate linear fits and 95% confidence intervals, respectively. L) Representative H&E and immunohistochemical staining for DDX21 and p16 in p16-positive and p16-negative HNSCC tissues. M) Kaplan–Meier analysis of overall survival in HPV-negative patients with HNSCC stratified by DDX21 expression into high (n = 34) and low (n = 54) groups. Survival distributions were compared using a two-sided log-rank test. The hazard ratio and 95% confidence interval were estimated using the Mantel-Haenszel method (HR = 0.49, 95% CI = 0.27–0.89; log-rank P = 0.032). Statistical significance was determined using a two-sided Wilcoxon matched-pairs signed-rank test in B, a two-sided paired Student’s *t*-test applied to ΔCt values in C, two-way ANOVA followed by Šídák’s multiple-comparisons test in D and F, two-sided unpaired Student’s *t*-tests in E and G, and two-sided Mann–Whitney *U* tests in H and I. Correlations in J and K were assessed using two-sided Pearson correlation tests. **P* < 0.05, ***P* < 0.01, and ****P* < 0.001.

**Figure S3. Effects of DDX21 overexpression on irradiation-induced DNA damage and 53BP1 foci in HN30 cells.**

A,B) Representative neutral comet assay images and quantification of tail moments in HN30 cells expressing control (Flag) and DDX21-overexpressing (Flag–DDX21) at 0, 4, 8 and 12h after exposure to 10 Gy irradiation. C) Representative 53BP1 immunofluorescence images of control (Flag) and DDX21-overexpressing (Flag–DDX21) HN30 cells at the indicated times after 10 Gy irradiation. Scale bars, 5 μm. D,E) Quantification of cells containing more than five 53BP1 foci, expressed relative to the corresponding non-irradiated control (NT) (D) or normalized to the corresponding 0 h value (E). Data are presented as mean ± SD from n = 3 independent biological experiments. Statistical significance was determined by two-way ANOVA followed by Šídák’s multiple-comparisons test. **P* < 0.05, ***P* < 0.01, ****P* < 0.001, and *****P* < 0.0001. NT, non-irradiated control.

**Figure S4. Changes in chromatin accessibility and H3K9me3 levels in DDX21-overexpressing HNSCC cells following irradiation.**

A,C) Representative MNase digestion patterns of chromatin isolated from control (Ctrl) and DDX21-overexpressing (OE) CAL27 and HN30 cells, respectively, at the indicated times after 10 Gy irradiation. Mono-, di-, tri-, and tetranucleosomal DNA fragments are indicated. B,D) Corresponding densitometric profiles of the MNase-digested DNA fragments in A and C, respectively. E) Representative mono-/trinucleosome ratios derived from A–D, providing a descriptive assessment of chromatin accessibility during the early irradiation response. F,G) Immunoblot and densitometric analyses of H3K9me3 in Ctrl and OE CAL27 and HN30 cells, respectively. H3K9me3 levels were normalized to total H3. H,J) Representative H3K9me3 immunofluorescence images of control (Flag) and DDX21-overexpressing (Flag–DDX21) CAL27 and HN30 cells, respectively. I,K) Quantification of the mean H3K9me3 fluorescence intensity in H and J, respectively. Scale bars, 5 μm. Data in F, G, I, and K are presented as mean ± SD from *n* = 3 independent biological experiments. Statistical significance was determined by two-way ANOVA followed by Šídák’s multiple-comparisons test. **P* < 0.05, ***P* < 0.01, ****P* < 0.001, and *****P* < 0.0001. NT, non-irradiated control.

**Figure S5. Effects of DDX21 expression on p38–MK2 signaling and caspase-3 cleavage following irradiation.**

A–C) Densitometric quantification of p-p38/p38, p-MK2/MK2, and cleaved caspase-3 (CC3)/caspase-3 in control and DDX21-overexpressing CAL27 cells following 0 or 10 Gy irradiation. D–F) Corresponding quantification in control and DDX21-overexpressing HN30 cells. G–I) Quantification in CAL27 cells expressing control shRNA (shCtrl) or either of two DDX21-targeting shRNAs (sh1 and sh2). J–L) Corresponding quantification in HN30 shCtrl, sh1, and sh2 cells. Data are presented as mean ± SD from *n* = 3 independent biological experiments. Statistical significance was determined by ordinary two-way ANOVA followed by Šídák’s multiple-comparisons test. **P* < 0.05, ***P* < 0.01, ****P* < 0.001, and *****P* < 0.0001.

**Figure S6. Effects of DDX21 depletion on irradiation-induced γH2AX levels in HNSCC cells.**

A,B) Immunoblotting and densitometric quantification of DDX21 and γH2AX in control and DDX21-depleted CAL27 and HN30 cells, respectively, following irradiation at the indicated doses. C,D) Immunoblot and densitometric analyses of DDX21 and γH2AX in control and DDX21-depleted CAL27 and HN30 cells, respectively, at the indicated recovery times following 10 Gy irradiation. γH2AX levels were normalized to α-tubulin. Data are presented as mean ± SD from n = 3 independent biological experiments. Statistical significance was determined by two-way ANOVA followed by Šídák’s multiple-comparisons test. **P* < 0.05, ***P* < 0.01, ****P* < 0.001, and *****P* < 0.0001. NT, non-irradiated control.

**Figure S7. Effects of DDX21 depletion on irradiation-induced DNA damage and 53BP1 foci in HN30 cells.**

A) Representative neutral comet assay images of HN30 cells expressing control shRNA (shCtrl) or DDX21-targeting shRNA (sh1) at the indicated times after 10 Gy irradiation. B) Quantification of tail moments in A. C) Representative 53BP1 immunofluorescence images of HN30 shCtrl and sh1 cells at the indicated times after 10 Gy irradiation. Scale bars, 5 μm. D,E) Proportions of cells containing more than five 53BP1 foci, expressed relative to the corresponding non-irradiated control (D) or normalized to 0 h (E). Data are presented as mean ± SD from *n* = 3 independent biological experiments. Statistical significance was determined by two-way ANOVA followed by Šídák’s multiple-comparisons test. **P* < 0.05, ***P* < 0.01, ****P* < 0.001, and *****P* < 0.0001. NT, non-irradiated control.

**Figure S8. Changes in chromatin accessibility and H3K9me3 levels in DDX21-depleted HNSCC cells following irradiation.**

A,C) Representative MNase digestion patterns of chromatin isolated from CAL27 and HN30 cells expressing control shRNA (shCtrl) or DDX21-targeting shRNA (sh1), respectively, at the indicated times after 10 Gy irradiation. Mono-, di-, tri-, and tetranucleosomal DNA fragments are indicated. B,D) Corresponding densitometric profiles of the MNase-digested DNA fragments in A and C, respectively. E) Representative mono-/trinucleosome ratios derived from A–D, providing a descriptive assessment of chromatin accessibility. F,G) Immunoblot and densitometric analyses of H3K9me3 in CAL27 and HN30 cells expressing shCtrl or either of two DDX21-targeting shRNAs (sh1 and sh2), respectively. H3K9me3 levels were normalized to total H3. H,J) Representative H3K9me3 immunofluorescence images of shCtrl and sh1 CAL27 and HN30 cells, respectively. I,K) Quantification of the mean H3K9me3 fluorescence intensity in H and J, respectively. Scale bars, 5 μm. Statistical significance was determined by two-way ANOVA followed by Šídák’s multiple-comparisons test. **P* < 0.05, ***P* < 0.01, ****P* < 0.001, and *****P* < 0.0001. NT, non-irradiated control.
